# *In-Vivo* Efficacy of Chloroquine to Clear Asymptomatic Infections in Mozambican Adults: A Randomized, Placebo-controlled Trial with Implications for Elimination Strategies

**DOI:** 10.1038/s41598-017-01365-4

**Published:** 2017-05-02

**Authors:** Beatriz Galatas, Lidia Nhamussua, Baltazar Candrinho, Lurdes Mabote, Pau Cisteró, Himanshu Gupta, Regina Rabinovich, Clara Menéndez, Eusebio Macete, Francisco Saute, Alfredo Mayor, Pedro Alonso, Quique Bassat, Pedro Aide

**Affiliations:** 1 Centro de Investigação em Saúde de Manhiça (CISM), Maputo, Mozambique; 2 ISGlobal, Barcelona Ctr. Int. Health Res. (CRESIB), Hospital Clínic - Universitat de Barcelona, Barcelona, Spain; 3 National Malaria Control Programme (NMCP), Ministry of Health, Maputo, Mozambique; 4 National Directorate of Health, Ministry of Health, Maputo, Mozambique; 5 ICREA, Pg. Lluís Companys 23, 08010 Barcelona, Spain

## Abstract

Recent reports regarding the re-emergence of parasite sensitivity to chloroquine call for a new consideration of this drug as an interesting complementary tool in malaria elimination efforts, given its good safety profile and long half-life. A randomized (2:1), single-blind, placebo-controlled trial was conducted in Manhiça, Mozambique, to assess the *in-vivo* efficacy of chloroquine to clear plasmodium falciparum (*Pf*) asymptomatic infections. Primary study endpoint was the rate of adequate and parasitological response (ACPR) to therapy on day 28 (PCR-corrected). Day 0 isolates were analyzed to assess the presence of the *PfCRT*-76T CQ resistance marker. A total of 52 and 27 male adults were included in the CQ and Placebo group respectively. PCR-corrected ACPR was significantly higher in the CQ arm 89.4% (95%CI 80–98%) compared to the placebo (p < 0.001). CQ cleared 49/50 infections within the first 72 h while placebo cleared 12/26 (LRT p < 0.001). The *PfCRT*-76T mutation was present only in one out of 108 (0.9%) samples at baseline, well below the 84% prevalence found in 1999 in the same area. This study presents preliminary evidence of a return of chloroquine sensitivity in Mozambican *Pf* isolates, and calls for its further evaluation in community-based malaria elimination efforts, in combination with other effective anti-malarials. *Trial registration:* www.clinicalTrials.gov NCT02698748.

## Introduction

In recent years, interest in the potential use of Chloroquine (CQ) has re-emerged, partly on account of the observation that the prevalence of molecular markers associated with CQ resistance among circulating parasites has decreased after discontinuing the use of the drug. The emergence of CQ resistance was first documented in Southeast Asia and South America less than a decade after its introduction as first-line treatment and spread to the East of Africa and the rest of the continent by the end of the 1970’s^1–3^. In 2001 the World Health Organization (WHO) recommended artemisinin-based combination therapy (ACT) as the first line treatment for uncomplicated malaria in countries where *Plasmodium falciparum* (*Pf*) malaria had become resistant to CQ^4^, and since then the use of CQ for the treatment of *Pf* has progressively been abandoned.

Drug-resistant microorganisms are generally thought to suffer from a fitness cost associated with their drug-resistant trait^5^, inflicting them a disadvantage when the drug pressure reduces. Such finding implies that resistance might be reversible if drug use is discontinued. A point mutation in the 76^th^ position of the *P. falciparum* CQ-resistance transporter gene (*PfCRT*) from a lysine in sensitive parasites (K76) to a threonine in resistant parasites (76 T) has been associated with CQ-resistant *falciparum* malaria^6^. Analysis of the population frequencies of the mutations in *PfCRT* known to be associated with CQ resistance^7^ and assessment of *in vitro* activity of the drug^8, 9^ in Malawian parasite isolates have indicated that CQ resistance may revert to sensitivity within a decade of withdrawal of the drug^10^. Similar drops, have been shown in Zambia^11^, Tanzania^12^ or coastal Kenya (Kilifi)^13^, years after CQ had been withdrawn. Translation of *in vitro* sensitivity to *in vivo* efficacy has also since then been demonstrated in Malawi^14^, where CQ efficacy in patients has been confirmed as very high^14^, and its use as monotherapy in a clinical trial context -albeit not a WHO recommendation- has also been shown to be similarly efficacious to its combination with other partner drugs^15^.

In Mozambique, which borders in the west with Malawi, the national policy moved away from using CQ in 2003–4. A study conducted among children from Manhiça District in 1999 revealed a *PfCRT 76* *T* prevalence of 84%^16^. However, a recent study performed in Gaza Province showed also an important decline of *PfCRT* K76T prevalence from more than 90% in 2006 to around 30% in 2010^17^. A similar study conducted in a different part of the country also confirmed this tendency towards an increase of wild type parasites and thus CQ sensitivity^18^. The last available efficacy results for CQ derived from an *in vivo* study performed in Mozambique in the year 2001 showed that the day 28 efficacy was 47.1%^19^.

Altogether, these data suggest that, in the absence of drug pressure, CQ may be regaining sensitivity against *P. falciparum* in certain malaria-endemic settings of Sub-Saharan Africa. While this does not support the reintroduction of CQ as first line therapy in such settings at this point, it does suggest that, if proven sensitive in a given area, CQ could be considered as a complementary tool to interrupt transmission in the context of malaria elimination efforts.

With this rationale in mind, we conducted a randomized placebo-controlled single blinded clinical trial to assess the efficacy of CQ (vs. placebo) to treat asymptomatic infections among healthy adult Mozambican volunteers. We complementarily present prevalence estimates of the *PfCRT* K76T molecular marker of CQ resistance among parasites among the study population in 2015.

## Methods

The protocol for this trial and supporting CONSORT checklist, are available and annexed as supporting information.

### Study design

Between January and June 2015, a randomized, single-blinded, placebo-controlled trial was conducted in the district of Manhiça, southern Mozambique, to treat asymptomatic infections among healthy adults from the community. This study population was considered as the most ethical option for the first *in-vivo* study to take place since 2001, when CQ had been shown to be poorly efficaciuos. A placebo comparator was consequently used to accurately account for the effect that the high levels of immunity expected in the study population would have on natural parasite clearance of low parasitaemic infections^20^.

### Study site

The district of Manhiça counts with a demographic surveillance system (DSS) set up in 1998 by the *Centro de Investigação em Saúde de Manhiça* (CISM), which currently provides accurate demographic information on its *circa* 178,000 inhabitants. The region has two distinct seasons – a warm and rainy season from November to April, and a cooler and drier season the rest of the year. Malaria transmission is perennial but shows marked seasonality, with *Plasmodium falciparum (Pf)* being the predominant species, and *Anopheles funestus* the main vector. Study participants were selected from areas within Manhiça were clinical malaria in children was historical known to be high (above 40%) and asymptomatic infections in adults were expected in the community^21^.

### Screening and recruitment of study subjects

The study population comprised adult males with microscopically confirmed, asymptomatic malaria infections. In order to find these subjects, a random list was generated from the DSS databases, and individuals were visited in their household, and screened for malaria through finger-prick with an HRP2-based rapid diagnostic test (RDT) and a blood slide. Blood slides were only read at CISM’s laboratory in those cases found to be positive by the RDT (“pre-screened positive”), providing a final confirmation of malaria infection and density of parasitaemia. On the following day, such individuals were again visited at home, and only if they remained symptomless were offered to be included in the study. Study staff enquired about the presence of symptoms by asking a series of questions following a standardised clinical questionnaire. Asymptomatic malaria was defined as the absence of any proactively referred symptom of disease as referred by the individual, together with a documented axillary temperature <37.5 °C. Symptomatic patients, irrespective of being infected or not, were assessed by the study clinician and referred to the health system if needed. Females were not screened on account of the Mozambican Ethics Committee’s recommendation to avoid exposing to placebo malaria-infected women of child-bearing age which may be pregnant and thus prone to developing severe disease. Other inclusion criteria included *Pf* malaria infection with an asexual blood density ≥100 p/µL but <10,000 p/µL. Key exclusion criteria included presence of any other co-existing clinical condition or symptom that in the opinion of the recruiting physician would not allow the individual to be considered a “healthy” asymptomatic carrier; axillary temperature > = 37.5 °C; intake of any medication which may interfere with antimalarial efficacy or antimalarial pharmacokinetics, such as cotrimoxazole; history of hypersensitivity reactions or contraindications to CQ; and known HIV concomitant infection under antiretroviral treatment. Individuals aged 18 years or more satisfying the inclusion criteria were enrolled if they signed a detailed written informed consent. Individuals aged 16 were offered participation provided their legal guardians also assented for participation.

### Treatment

After the pre-screening visit (Day “−1”), a screening evaluation was conducted 24 hours later (day 0) to check all inclusion and exclusion criteria, with a particular emphasis on ensuring that symptoms had not appeared. Eligible patients were randomly assigned to receive CQ (Arm 1) or Placebo (PL; arm 2), according to a 2:1 (CQ:PL) scheme, so as to have more patients in the CQ arm to provide better estimates for its cure rates. Patients having received on day 0 a study drug, but confirmed by the blood slide to be malaria infection negative or <100 parasites/µL were considered protocol violations, and excluded from the analysis. A randomization list was generated using the online available randomizer software (http://www.randomizer.org/). Study staff carried the study medication and directly supervised the three-day long treatment. Chloroquine sulphate (Meriquine®; 250 mg; 150 mg of chloroquine base; Baroda, India) was administered at a total dose of 25 mg/kg (expressed as mg of CQ base per kg body weight, once daily during 3 consecutive days, following the schedule 10 mg/kg Day 1; 10 mg/kg day 2 and 5 mg/kg day 3; or four, four and two pills per day, respectively). A similarly-looking placebo not containing any active principle, and prepared at the department of pharmacology of the Hospital Clinic, Barcelona, Spain, was administered to those allocated this intervention following an identical scheme. All treatments were directly observed for a minimum of 30 min. Any subject vomiting during this observation period was re-treated with the same dose of either CQ or placebo, and observed for an additional 30 min. Repeated vomiting implied withdrawal from the study and the use of rescue treatment. Treatment of symptomatic malaria infections or rescue therapy in cases of early or late treatment failure followed national Mozambican guidelines and included the use of artemether-lumefantrine (AL)^22^.

### Evaluation

Follow-up visits were planned on days 1, 2, 3, 7, 14, 21 and 28 after enrolment, or at any time point should the enrolled individual develop any symptoms of sickness. Individuals who discontinued either study drug were excluded from the study. Vital signs and body temperature were assessed during each follow-up visit. Adverse events were recorded and assessed for severity and association with study medication.

Thick and thin Giemsa-stained blood slides were prepared prior to the administration of the drug and at every follow-up visit. Slides were examined by two independent microscopists and considered negative if no parasites were seen after examination of 200 oil-immersion fields in a thick blood film. Parasite density was estimated using the Lambaréné method^23^ which counts parasites against an assumed known blood volume. Density of *P. falciparum* was assessed from blood-spots collected in filter paper through real-time quantitative polymerase chain-reaction (qPCR) assay targeting 18S ribosomal RNA (rRNA)^24, 25^. Blood spots for PCR analysis were collected using 3M Whatman™ filter papers at baseline and at days 7, 14, 21 and 28, on the day of treatment failure or at any other unscheduled visit, and subsequently stored at 4 °C in plastic zip bags containing silica gel dessicant. PCR was performed centrally (Barcelona, Spain) for all cases of recurrent parasitaemia from day 7 onwards, or for whom evidence of parasite clearance had been documented, and including DNA extraction using a QIAamp DNA Mini Kit (Qiagen). The three polymorphic genetic markers MSP1, MSP2, and GluRP, were also investigated to distinguish recrudescence from new infections, according to WHO recommended procedures^26^. Recrudescence was defined as at least one identical allele for each of the three markers in the pre-treatment and post-treatment samples. New infections were diagnosed when all alleles for at least one of the markers differed between the two samples.

### Clinical trial outcomes

Treatment outcomes were classified on the basis of an assessment of the parasitological and clinical response to antimalarial treatment according to the latest WHO guidelines^27^. However, as all recruited subjects were asymptomatic at baseline, some of the standard clinical endpoints such as fever clearance time were not applicable. The primary efficacy outcomes were the PCR-corrected early treatment failure (ETF), late clinical failure (LCF), late parasitological failure (LPF) and adequate clinical and parasitological response (ACPR) at day 28. ACPR was defined as the absence of parasitemia at the end of the trial’s follow-up period (Day 28), regardless of axillary temperature, without having previously met any of the criteria for early and late treatment failure. In the PCR-adjusted analyses, patients with recurrent infection were considered ACPR if this was classified as a new infection. Secondary outcomes included 28 day-uncorrected ACPR (crude efficacy), time to parasite clearance, parasite clearance curve during the first 72 hours of follow-up, and prevalence of chloroquine-resistance conferring *PfCRT* K76T mutation in pre-treatment infections.

### Assessment of PfCRT K76T mutations

Purified DNA templates were amplified using 2720 Thermal Cycler (Applied Biosystems) by nested PCR amplification followed by Sanger sequencing for *PfCRT* gene. In brief, a first round of amplifications was performed in 25 μl reactions including 5 μl of template DNA, 0.5 μM of each forward (*PfCRT*_F-5′tttaggtggaggttcttgtctt-3′) and reverse (*PfCRT*_R-5′ atacttaattgaagaacaaatgattgga-3′) primers and 1x HOT FirePol Master Mix (Solis BioDyne; Cat. No. 04-27-00125). The template DNA was denatured at 95 °C for 15 min in a thermocycler, followed by 25 cycles of amplification (95 °C for 1 min, 52 °C for 45 sec, and 72 °C for 45 sec) and a final extension at 72 °C for 10 min. A reaction using 5 μl of PCR-grade water instead of template DNA was included as a negative control. For the nested amplification, 5 μl of the PCR product from the first amplification was used as the template in a PCR reaction (50 μl final volume) containing 0.5 μM of each forward (*PfCRT*_F) and reverse (*PfCRT*_N_R-5′ttggtaggtggaatagattctct-3′) primers and 1x HOT FirePol Master Mix (Solis BioDyne). The reaction volume was make up by PCR-grade water. The template DNA was denatured at 95 °C for 15 min in a thermocycler, followed by 35 cycles of amplification (95 °C for 30 sec, 50 °C for 45 sec, and 72 °C for 45 sec) and a final extension at 72 °C for 10 min. PCR products were run on 2% agarose (Invitrogen) gels in 1 × TBE buffer (Thermo Fisher Scientific) to determine the presence and size of the amplified DNA. PCR products were visualized using a UV trans-illuminator. Both PCR primer sets were also tested with human gDNA to check their specificity. Three positive (7G8, Dd2, and V1/S) controls with known *PfCRT*_76 T allele were also processed and amplified at the same time as the studied samples. Positive and negative controls were added in every run. The expected size of the nested PCR was 319bp covering amino acid positions 35 to 120 from *PfCRT* in the 3D7 strain. PCR products were quantified using EPOCH Biotech system. Approximately 1200ng of PCR products were sent to Genewiz, following safety instructions for the accurate shipment of PCR amplicons. PCR sequencing was performed in both directions using specific forward and reverse primers of studied genes. The variations in the test sequences were identified by sequence alignment against reference sequence of 3D7 (PF3D7_0709000) retrieved from PlasmoDB.

### Data management and statistical analysis

Data were recorded using standardized questionnaires specifically designed for this study, which were doubled-entered into a study-specific database created using open clinica software (OpenClinica Enterprise - Electronic Data Capture Software for Clinical Trials version 3.1.2, OpenClinica LLC, Waltham, MA, USA). Statistical analyses were performed with Stata 14 (Stata Corp, College Station, TX, USA), and the statistical significance level was set at 5%.

Safety outcomes were assessed in the intent-to-treat (ITT) population, which comprised all patients who received one or more doses of study medication and underwent at least one post-baseline safety assessment. Efficacy was calculated in the according-to-protocol population (ATP), which included all patients fulfilling the protocol eligibility criteria, who completed the three-day course of study medication, with no protocol violations, accomplishing the day-28 assessment and having an evaluable PCR in case of recurrent parasitaemia. Kaplan-Meier estimates were computed to estimate the cumulative proportion of treatment success until day 28; and Log-Rank tests used to compare the number of events in each arm. For such an analysis, losses to follow-up and study withdrawals were censored on the last day of follow-up. Cases with re-infections were also censored from the analysis on the day of detection. Finally, linear regressions and Likelihood Ratio Tests (LRT) of the log-transformed parasite densities during the first 72 hours of treatment were estimated to better characterize the parasite clearance curve during treatment between study groups.

Frequencies of mutations at *PfCRT*-76 alleles were calculated as the proportion of samples carrying the mutant form out of all samples processed. Samples carrying both wildtype and mutant forms, in which relative frequencies could not be determined, were excluded from the denominator and numerator^13^.

### Sample size calculations

Following WHO guidelines^28^, and assuming an unknown failure rate, a minimum sample of 50 patients was considered necessary to provide a meaningful evaluation of *in vivo* efficacy, regardless of the underlying rates of failure. For the placebo group, the proportion of parasites that are eliminated naturally by the host’s immune system after 28 days in Manhiça is unknown, but expected to be less than one percent^29–31^. Thus, the sample size for the control group needed not be the same size as the treatment group in order to detect a significant difference in prevalence. If 25 controls were recruited (placebo arm), then, even if half of the controls were to clear their infections, we will still achieve a p-value of less than 0.001 from a Chi-square test with one degree of freedom (Fisher’s exact test would also yield a p-value less than 0.001). Therefore, our objective was to recruit 75 participants total, i.e. 50 to receive CQ and 25 to receive PL.

### Ethical considerations

This protocol, consent forms and questionnaires were approved by the CISM local ethics committee, the Ethics Committee of the Hospital Clínic of Barcelona (HCB/2015/0122), the National Bioethics Committee of Mozambique (CNBS; Ref:173/CNBS/13) and the Mozambican pharmaceutical department (Ref./N°4110/380/DF2014) before their implementation. The methodology used in this study was performed in accordance with the relevant regulations and guidelines from all committees. All participants signed an Informed consent form prior to the initiation of any study related activities. All samples for the separate analysis of trends in CQ resistance were obtained under an informed consent, and after individual ethical approvals for each of the parent studies by CNBS. The trial was registered on January the 13^th^, 2015, at www.ClinicalTrials.gov (NCT02698748).

## Results

### Trial profile and baseline characteristics

**Figure 1:**
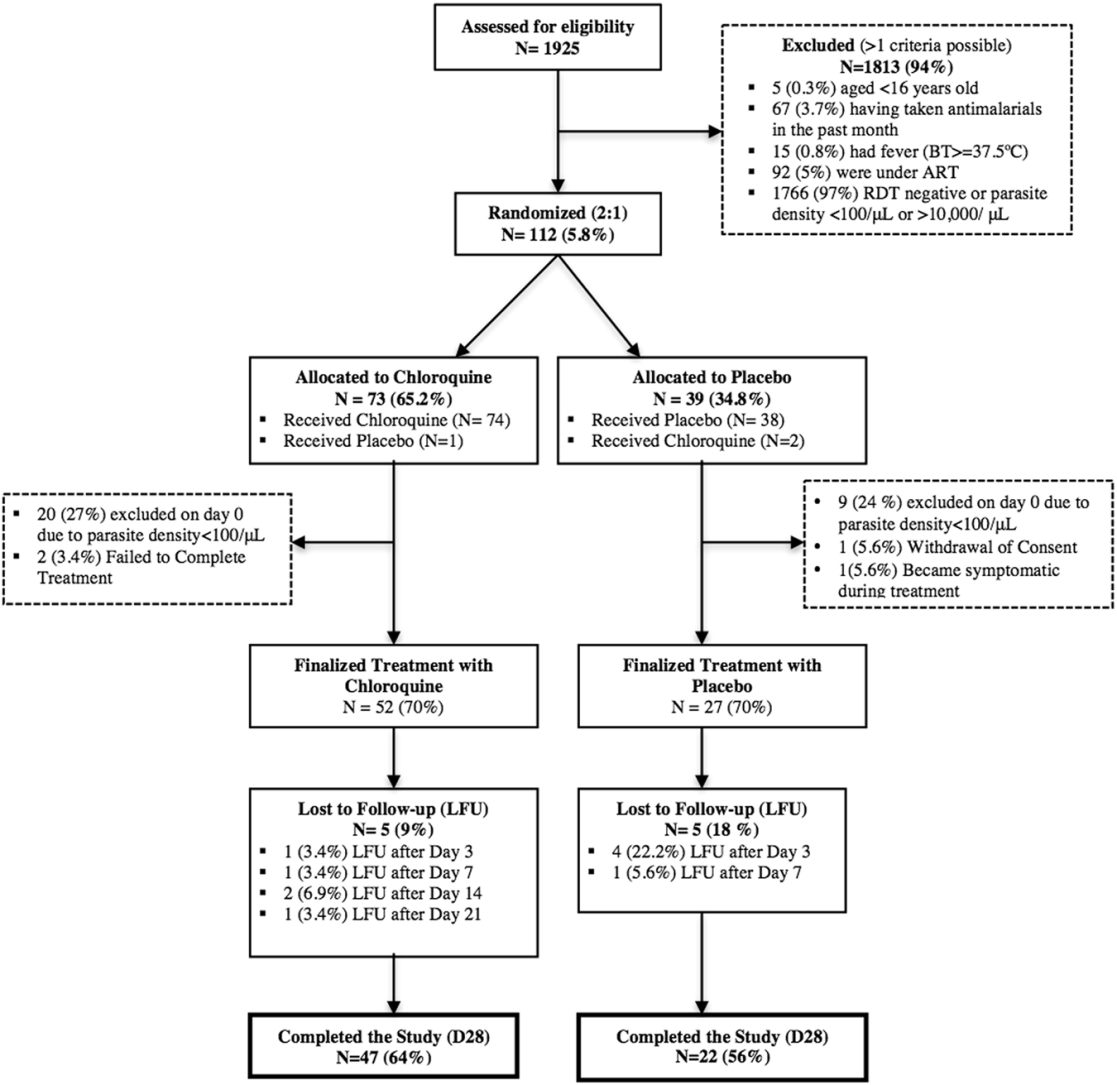
Consort flow diagram of study participants.

A total of 1925 adult males were approached and pre-screened (Day −1) for symptomatology and malaria infection. Of those, 112 (5.8%) fulfilled *a priori* inclusion criteria and were randomized (Day 0) to receive either CQ (n = 73; 65.2%) or PL (n = 39; 34.8%). Median age of randomized individuals was 21.5 years (IQR 17–35). No significant differences were observed between subjects randomized to receive either CQ or PL (Table 1). Main reasons for exclusion was the absence of malaria parasites at pre-screening and/or the concomitant use of antiretroviral or antimalarial drugs. The ITT and ATP populations included 112 and 79 individuals, respectively. One patient that should have received CQ was given by mistake PL, and conversely, two patients that should have received PL were treated with CQ, which resulted in 74 participants in the CQ arm and 38 in the PL. In those three cases, subjects continued with the same treatment for the entire 3 days. Two patients in the CQ group did not complete the entire 3-day long treatment. A single patient in the PL group had to be withdrawn from the study and given rescue treatment after developing clinical symptoms. 52 patients finalized the CQ treatment, and 27 finalized the PL. Of these, 47 subjects allocated to receive CQ (64%) and 22 to PL (56%) concluded with all available information the 28 day long follow-up (Fig. 1).

**Table 1:** Baseline characteristics of participants at enrolment.

Variable	Treatment Group			p-value^3^
	Placebo	CQ	Total	
Total Number of Participants on Day 0	38	74	112	
Age (years)^1^	26 (14) [38]	29 (16) [64]	28 (15) [102]	0.222
Body Temperature (°C)^1^	36.5 (0.4) [38]	36.5 (0.3) [70]	36.5 (0.3) [108]	0.732
Weight (kg)^1^	55.3 (10.6) [37]	58.6 (8.8) [71]	57.5 (9.5) [108]	0.088
Parasite density (p/μL)^2^	488.3 (864.9) [34]	534.0 (1010.2) [62]	517.3 (952.5) [96]	0.821
PCR Parasitemia (p/μL)^2^	185.5 (349.9) [37]	169.4 (332.0) [71]	174.8 (336.7) [108]	0.818

1: Arithmetic Mean (SD) [n] 2: Geometric Mean (SD) [n]. 3: T-test.

### Safety

In terms of tolerability and safety of the interventions, the symptoms reported during the three-day treatment course did not significantly vary between study arms (Table 1). No serious adverse events occurred, and all AEs were very mild and deemed not related with the interventions except for a case of mild pruritus reported from one patient in the CQ group.

### Efficacy

**Figure 2:**
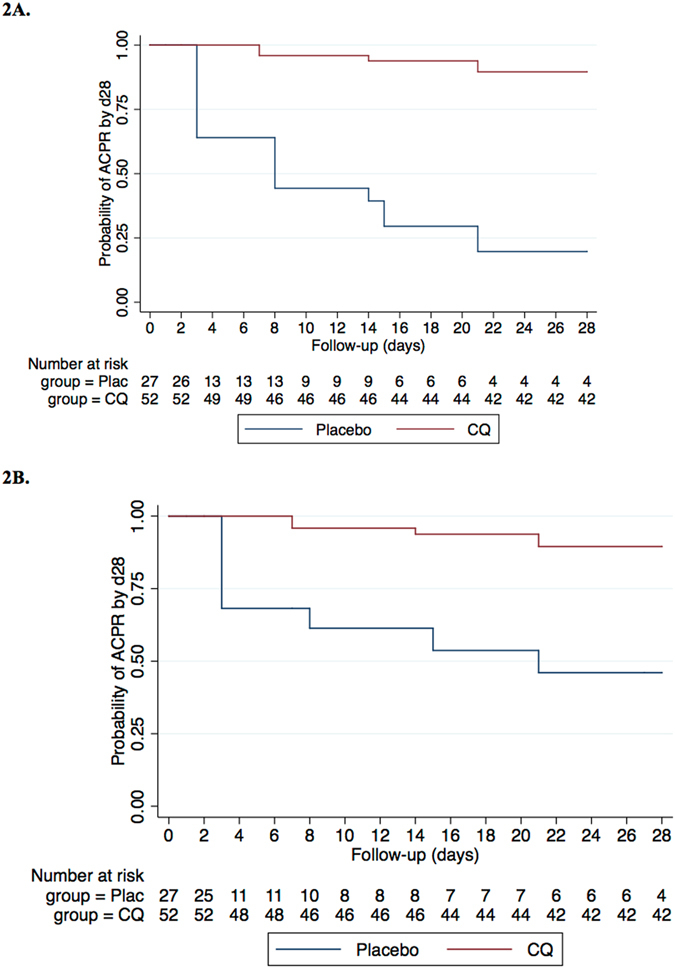
PCR uncorrected (**2A**) and PCR-corrected (**2B**) Kaplan Meier curves showing the treatment success cumulative proportion for each study arm until day 28 in the ATP population. Cumulative risks of failure were computed for day 28 and Log-Rank tests used to compare the number of events that occurred in each arm. PCR-corrected curves censored participants who experienced a reinfection on the day it was detected.

The day-28 PCR-uncorrected cure rate (i.e., the proportion of patients with ACPR, ATP population, without correcting by PCR) was 89.4% (42/47; 95% CI 80.5–98.2) for CQ, and 18.1% (4/22; 95% CI 2.1–34.3) for PL (p < 0.001). PCR-correction did not change the D28 ACPR for CQ, which remained 89.4% (42/47; 95% CI 80.5–98.2) given that the only new infection identified in this group was lost to follow-up after day 21; but increased that of PL (28.6%; 4/14; 95% CI 4.9–52.2), as 8 of the treatment failures proved to be new infections, and were consequently excluded from the analysis (Table 2). Figure 2 illustrates the Kaplan Meier curves showing the treatment success cumulative proportion for each treatment until day 28, both for the PCR uncorrected (2a) and PCR-corrected (2b) in the ATP population.

**Table 2:** Treatment outcomes on day 28, according to treatment received (Placebo or chloroquine).

Variable	Placebo	CQ	p value
	N = 27	N = 52	
ACPR^a^ (uncorrected) n	4	42	<0.001*
ETF^b^ n	11	0	<0.001*
LCF^c^ n	0	0	NA
LPF^d^ n	9	7	0.037**
New infections (with PCR) n	8	1	0.034
Recrudescences (with PCR) n	1	1	
No treatment outcome: loss to follow-up or missing or inconclusive PCR results	9	12	NA
ATP^e^ day-28 efficacy (PCR-uncorrected) n/N (95%CI) by optic microscopy	4/22 (18.2%) [2.1–34.3]	42/47 (89.4%) [80.5–98.2]	<0.001*
ATP day-28 efficacy (PCR-corrected) n/N (95%CI)	4/14 (28.6) [4.9–52.2]	42/47 (89.4%) [80.5–98.2]	<0.001*

^a^ACPR:adequate clinical and parasitological response; ^b^ETF: early treatment failure; ^c^LCF: late clinical failure; ^d^LPF: late parasitological failure; ^e^ATP: According to protocol; NA: not applicable. *Fisher’s exact test; **Chi-squared test.

### Parasite clearance within first 72 hours

**Figure 3:**
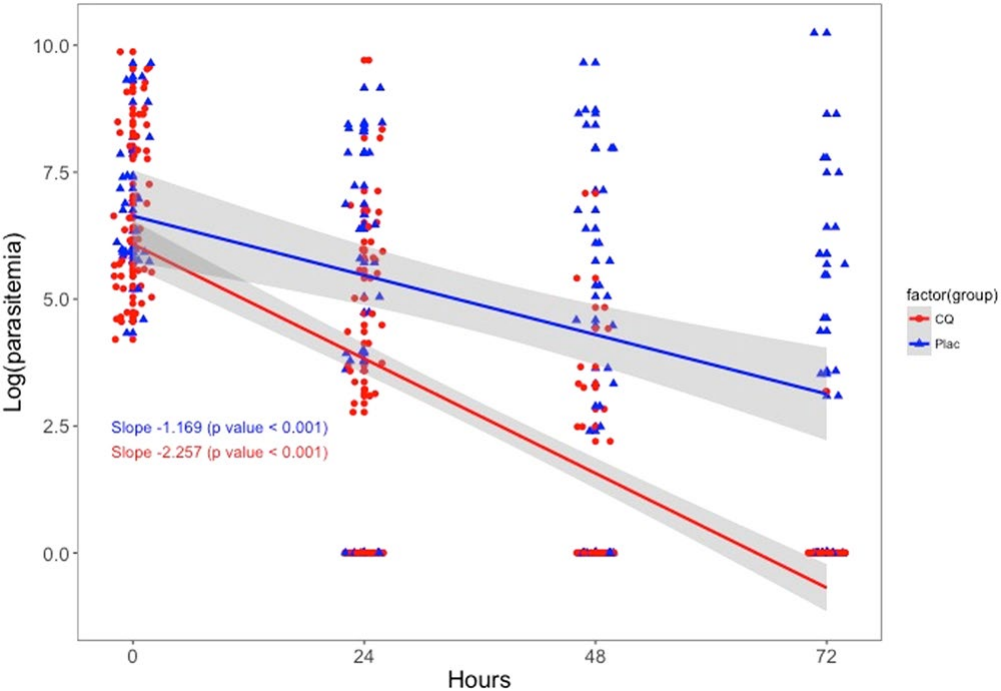
Linear regressions fitted to the log-transformed parasite densities during the first 72 hours after first-dose administration.

Only one (2%) of the 50 participants in the CQ arm still presented an infection (24 p/μL) 72 hours after the administration of the first dose. Conversely, 14 (54%) of the 26 individuals in the placebo group were infected at the same point in time, with parasite densities ranging from 22 to 28,089 p/μL. Finally, the linear regressions fitted to the log-transformed parasite densities at 0, 24, 48 and 72 hours revealed significantly different rates of parasite clearance (LRT of interaction p-value = 0.02) between study arms (Fig. 3).

### PfCRT 76 mutations among isolates from the clinical trial

**Figure 4:**
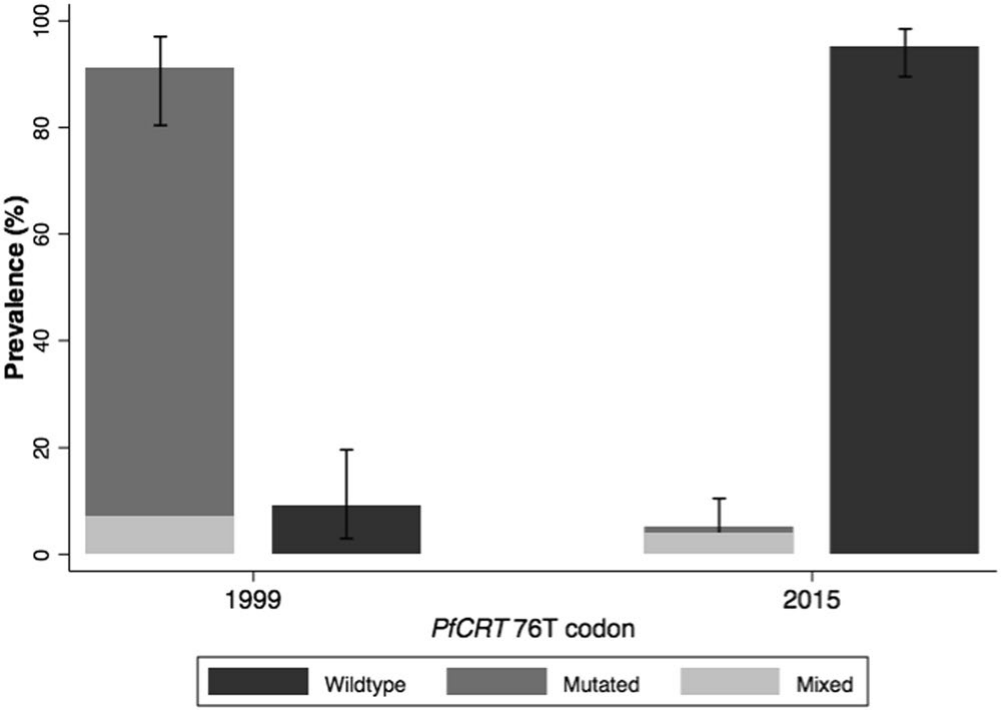
Prevalence of *PfCRT* K76 mutated, wildtype and mixed parasites from samples collected in 1999 and 2015. Samples were genotyped using restriction fragment length polymorphism in 1999 and bi-directional DNA sequencing in 2015.

Among 110 samples, 108 samples were used to assess the presence of *PfCRT* K76T mutation by bi-directional DNA sequencing. Further, these sequences were aligned, using NCBI-Blast online tool against the reference sequence of 3D7 (PF3D7_0709000) retrieved from PlasmoDB. Three positive control data were in accordance with existing data. Only one of the 108 (0.9%) samples analyzed was found to have the mutant allele (Fig. 4). Four additional samples (3.7%) had mixed infection, and wild type allele frequency was 95.4% (103/108).

## Discussion

In the continuum of strategies governing the transition towards malaria elimination^32^, there has been renewed interest in the innovative reappraisal of CQ as an antimalarial to be used in community-based campaigns. However, evidence is needed to demonstrate that CQ-based regimens will be effective over a wide geographic area where malaria elimination interventions will be implemented. This study presents a return of chloroquine sensitivity in Mozambique approximately 12 years after its use was interrupted. In the last decade, the presence of resistant parasites has dropped from 84% in 1999^16^ to 0.9% in 2015. Additionally, in 2015 chloroquine successfully cleared 89% of asymptomatic infections among a population of male adults from an endemic area, when compared to infections treated with placebo. The rate of parasite clearance was also significantly faster among chloroquine-treated infections compared to those left untreated and, as expected, there were no adverse events associated to the medications. This study provides the first evidence to support the idea that CQ could play a role, on its own or in combination with other drugs or tools, in the fight against malaria in Mozambique.

The drug efficacy observed in the present clinical trial is promising despite falling below the WHO recommended 95% efficacy rate for antimalarial drugs^28^. The methodology used to assess efficacy in this study was unusual for an anti-malaria efficacy study, thus conditioning the interpretation of its results to the advantages and disadvantages of the clinical trial procedures followed. Comparing chloroquine versus placebo allowed to test the hypothesis that chloroquine was as inefficacious as not providing any treatment at all. By having an untreated control, this methodology allowed assessing whether the cleared infections observed in the chloroquine group were solely due to the body’s natural capacity to eliminate the parasite reservoir rather than due to the effect of the drug. The placebo group of this study revealed that approximately 18% of the low-parasitemia infections are naturally cleared 28 days after they have been identified, a larger proportion compared to what has previously been reported^23–25^. In addition, only one individual developed clinical symptoms among all untreated asymptomatic participants. This is therefore an unprecedented opportunity for further exploration of the relatively unknown symptom-less malaria infection^33^, which will be performed in detail and presented in a separate article.

Considering the low *in vivo* efficacy of chloroquine detected in the same study area prior to this clinical trial^19^, the study only included asymptomatic infections to avoid treatment failures that could trigger complications to the study participants. This study population implies several limitations inherent to the nature of asymptomatic infections. First, the high levels of existing immunity in the study population may have augmented the efficacy of the drug, although this was controlled for in the analysis by observing the natural parasite clearance rate in the placebo group with the same levels of immunity^20^. Unfortunately, the study design did not consider regular estimations of parasite densities during the first days of follow-up, which limited our capacity to accurately estimate parasite reduction rates (PRR) in both treatment groups as a surrogate of drug efficacy^34^. Second, low parasitaemic infections (approximately 500 p/μL) could have potentiated drug efficacy, which needs to be assessed further in future studies involving clinical malaria cases with higher loads of parasite biomass. The evaluation of drug efficacy in low parasite density infections carried additional challenges, particularly when performing molecular analysis to comply with WHO standard analysis procedures^35^. Due to low parasitaemia, the sensitivity of the genotyping techniques was low, and therefore PCR-Corrected analysis was considered biased. As a result, PCR-uncorrected analysis should be considered as a more precise way of analyzing the efficacy of chloroquine in this particular study population. The standard PCR-Corrected analysis was also limited by the very nature of the control group, which consisted of natural, untreated, asymptomatic infections. Participants in the placebo group were consequently vulnerable to reinfections and parasite density oscillations around the microscopy detection threshold that could be interpreted as recrudescences or reinfections rather than as a chronic low parasitemic infection^4^.

Despite all of the above, the high *in vivo* efficacy found in Southern Mozambique through this clinical trial aligns well with the findings obtained molecularly that detected a sharp decline in the prevalence of drug-resistant parasites between 1999 (84%) and 2015 (0.9%) in the same area. Such a shift in the parasite population after the interruption of CQ use has been observed in several countries in Africa^10, 12, 13, 36^. This reversal to the wild type form of the *PfCRT* gene indicates that parasites carrying the mutant *PfCRT* may have a substantial fitness cost in the absence of CQ, thus leading to their decline in frequency once drug pressure is removed^14^. In some instances, loss of fitness may be associated with the development of compensatory mechanisms, leading to persistence of the mutant parasite in the population despite the discontinuation of the drug. This feature may explain, at least in part, the persistence of *PfCRT*-76 mutant^37–39^, even if CQ had not been used in these areas for many years. There are a series of additional factors that may be involved in the presence of phenotypic CQ resistance – such as PfMDR1 mutations^40^ – that were not assessed in this study and could lead to an underestimation of the real molecular resistance profile in the area. These factors could be the cause of remaining CQ resistance unrelated to the presence of *PfCRT*, and should be considered when monitoring molecular resistance in the future.

Overall, the encouraging results from this first exploratory trial and molecular analysis set the scene for subsequent studies to continue exploring the efficacy of CQ in clinical cases with higher parasitaemia, before any final recommendations are made with regards to the use of this drug in Mozambique. It could be argued that exposing children with clinical malaria to a drug that has only shown partial efficacy to clear infections in semi-immune adults with low-density, may be hard to justify based on the presented data. Indeed, this population, with allegedly lower levels of acquired immunity to malaria due to their age, may be more vulnerable to the infection, and at higher risk of responding poorly to a partially effective drug. Thus, as a next step, we aim to continue testing the *in vivo* efficacy of CQ among adults, but this time enrolling only those with clinical symptomatology derived from their infections, and higher parasite densities on admission. Only if chloroquine shows good efficacy in this population, we will deem it possible to start evaluating CQ’s real life efficacy among infected sick children.

In the context of malaria elimination, CQ exhibits two conditions that make it attractive for elimination campaigns: 1) It has been demonstrated to have an excellent safety profile, allowing for its use in all age groups including pregnant women and young children; and 2) Its relatively long elimination half life (t1/2 = 1–2 months)^41^ can provide a long post-treatment prophylactic effect. Thus, CQ could be a drug of choice in malaria elimination efforts such as in MDA campaigns, in combination with a highly effective anti-malarial. It could also be used in similar contexts for those populations who cannot receive ACTs, such as pregnant women in the first trimester, or very young children, as the safety of CQ in such populations has widely been demonstrated. Finally, through this innovative use of CQ, the drug pressure exerted to the circulating parasites would be minimal, and risk for re-introduction of resistant mutations would be very low.

## Conclusion

This study shows that CQ could play a role, on its own or in combination with other drugs or tools, as part of malaria elimination efforts at the population level, both on account of its therapeutic efficacy but more importantly by means of its chemoprophylactic activity and safety profile.
